# Changes in attitudes, risky practices, and HIV and syphilis prevalence among female sex workers in Brazil from 2009 to 2016

**DOI:** 10.1097/MD.0000000000009227

**Published:** 2018-05-25

**Authors:** Célia Landmann Szwarcwald, Wanessa da Silva de Almeida, Giseli Nogueira Damacena, Paulo Roberto Borges de Souza-Júnior, Orlando da Costa Ferreira-Júnior, Mark Drew Crosland Guimarães

**Affiliations:** a Health Information Laboratory, Institute of Communication and Scientific and Technological Information in Health, Oswaldo Cruz Foundation; b Institute of Biology, Federal University of Rio de Janeiro, Rio de Janeiro; c Federal University of Minas Gerais, Belo Horizonte, Minas Gerais, Brazil.

**Keywords:** Brazil, FSW, HIV and syphilis prevalence, RDS, temporal changes

## Abstract

**Background:**

The present study aims at investigating the progress made toward controlling the human immunodeficiency virus (HIV)/AIDS epidemic among female sex workers (FSW) from 2009 to 2016.

**Methods:**

The baseline of respondent-driven sampling (RDS) study among FSW was carried out in 2009, in 10 Brazilian municipalities. In 2016, information on FSW were collected in 12 municipalities. The analyses took into account the dependence among observations, resulting from the recruitment chains, and the unequal probabilities of selection, resulting from the different network sizes. We analyzed changes in attitudes and risky behavior practices as well as variations in HIV and syphilis prevalence based on the comparison of 95% confidence intervals for each estimate.

**Results:**

Information on 2523 (2009) and 4245 (2016) FSW were analyzed. Commercial sex debut shifted to younger ages: while in 2009 the proportion of women who started sex work under 18 years old was 28.3%, in 2016 this percentage rose to 38.3%. The proportion of FSW affiliated to a nongovernmental organization (NGO) in defense of their rights (14.0%), in 2009, decreased to 7.8%, in 2016, as well as the proportion of FSW who received counseling on sexually transmitted infections (STI) in the past 6 months, from 47.5% to 24.4%. Relevant improvements were found for HIV testing in the last 12 months (from 20.3% to 39.3%). The proportions of those who were never tested for syphilis dropped from 57.9% to 48.5%. However, an opposite decreasing trend was found for the Pap smear examination in the last 12 months, decreasing from 43.6% to 31.5%. Regular condom use with clients significantly increased in the period. Regarding HIV prevalence, the 5% level was sustained and no significant differences were found, but syphilis prevalence was found to be more than 3 times higher in 2016 (8.5%) than in 2009 (2.4%).

**Discussion:**

Many are the challenges to be faced in attempting to reverse the upward trend of syphilis among FSW in Brazil. Despite the progress in condom distribution free of charge, it is necessary to increase awareness campaigns, emphasize the use, reaffirm STI counseling, and reiterate the need of regular syphilis screening in this key population group.

## 1 Introduction

Since the beginning of the human immunodeficiency virus (HIV)/acquired immune deficiency syndrome (AIDS) epidemic in Brazil, in the early 1980s, HIV prevalence has remained at levels lower than 1% in the general population, and it has been largely “concentrated,” with higher rates among most-at-risk population groups.^[1]^

In this concentrated scenario, groups at higher risk for HIV infection play a key role in the dynamics of the epidemics. The spread of HIV infection is influenced by the nature and intensity of interactions between the most-at-risk groups and the general population.^[2,3]^ Through mathematical modeling, it has been shown that in epidemics with low potential reproduction rates, modest interventions in high risk groups can significantly reduce HIV incidence.^[4,5]^

In Brazil, injecting drug users (IDUs), men who have sex with men (MSM) and female sex workers (FSW) are considered the most-at-risk groups for HIV infection.^[1]^ Other population groups considered key elements in the spread of HIV infection are those that serve as bridges between the general population and vulnerable groups, such as clients of FSW.^[6–8]^

Conducting studies on subpopulations of HIV risk with conventional sampling strategies is complicated.^[9]^ In addition to the small population size, these groups are in general linked to stigmatized behaviors or illegal activities and are considered to be hard to reach populations.^[10]^ Carrying out population-based studies in order to generate representative estimates of HIV prevalence among FSW, broken down by variables of interest, such as age, educational level, and place of work, would require very large sample sizes and would not be feasible due to operational and cost difficulties.^[11,12]^

It is estimated that FSW represent 0.8% of the Brazilian female population from 15 to 49 years of age, accounting for a half million women, approximately.^[13]^ Prostitution is not considered a crime under the National Constitution, except if minors are involved.^[14]^

In Brazil, efforts are being made to develop a series of cross-sectional studies to monitor risk behavior practices of most-at-risk populations for HIV infection at the national level. In 2006, the Department of Prevention, Surveillance and Control of Sexually Transmitted Infections, HIV/AIDS and Viral Hepatitis (acronym in Portuguese—DDAHV) of the Brazilian Ministry of Health (MoH), promoted, in partnership with the Centers for Disease Control and Prevention (CDC), University of California San Francisco, University of Tulane, and Oswaldo Cruz Foundation (FIOCRUZ), the transfer of sampling methodology to access hard to reach population groups.^[1]^ In 2009, research projects were carried out in 10 Brazilian cities among 3 population subgroups: MSM,^[15]^ FSW,^[16]^ and drug users (DU)^[17]^; using respondent-driven sampling (RDS) as the recruitment method.^[18]^ Following the DDAHV commitment of HIV surveillance among FSW, a second cross-sectional survey using the same methodology was carried out in 2016 and built on the 2009 survey, defined as the baseline for future comparisons.

To analyze data collected by RDS, specific methods of analysis were developed, considering the structure of dependence of the observations and the unequal probabilities of participant selection.^[19–21]^ The present study aims at investigating the progress made toward controlling the HIV/AIDS epidemic and changes in attitudes and risk behavior practices for sexual transmitted infections (STI) among FSW from 2009 to 2016.

## 2 Methods

### 2.1 The baseline study, 2009

The baseline RDS study among FSW was carried out in 2009, aimed at estimating prevalence of HIV and syphilis and establishing knowledge, attitudes, and practices related to HIV infection and other STI in this population group.

To conduct the research, the DDAHV selected 10 Brazilian municipalities based on the importance of the local AIDS epidemic. The sample size (2500 women) was calculated to estimate a 6% HIV prevalence, with a 95% confidence interval and 2-tailed error of 1.5%, considering a design effect of 1.5. In each of the municipalities, the attempt was made to distribute the sample proportionally to the municipality population, while setting a minimum sample of 100 women (Table 1).

**Table 1:**
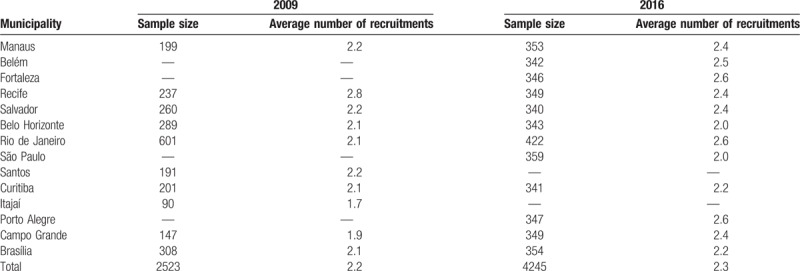
Sample size and average number of participant recruitments by municipality (FSW studies, 2009 and 2016).

The eligibility criteria were the following: women (biologically determined at birth) aged 18 years or older; who reported commercial sex at least once in the previous four months in each municipality, to assure recent sex work activity in the city; and accepted to participate in the study and signed the informed consent form. In addition, they should present a valid coupon and should not have participated in the study previously.

The study consisted of a preliminary interview—to verify the eligibility criteria and to characterize the network size and work venue; a self-completed questionnaire; and rapid tests for syphilis and HIV. In all 10 municipalities, the fieldwork was carried out in health services. Before data collection, a qualitative preparatory investigation was performed in each municipality to facilitate the study implementation taking into account their respective prostitution characteristics.

In each municipality, 5 to 10 initial participants, named seeds, were selected with different sociodemographic and sex work characteristics. The initial focus was on well-connected FSW in the community and who reported extensive social networks during the preparatory survey.

Each seed received 3 coupons to distribute to other sex workers within her social network. The recruits of the seeds in the survey were considered the first wave of the study. After participating in the interview, each participant received 3 additional coupons to distribute to their peers and this process was repeated until the sample size was achieved in each site.

RDS requires a system of primary and secondary incentives. The primary incentive in this study was a gift (makeup product), payment for lunch and transportation in addition to a reimbursement for their time lost from work (approximately US$15.00). The secondary incentive was a payment of US$10.00 for each recruited person who participated in the study.

The questionnaire was completed directly on a computer using ACASI (audio computer-assisted self-interview) and included the following aspects: sociodemographic and sex work characteristics; access to preventive activities; access and utilization of health services; knowledge, attitudes, and risky practices for HIV and other STI; history of HIV and syphilis testing; sexual behavior with steady partners and clients; violence and discrimination; and illicit drugs and alcohol use.

Rapid tests were performed using capillary blood samples drawn from participants according to the MoH protocols, including pre and post-test counseling. Participants who tested positive were referred to public health services for appropriate follow-up according to the MoH guidelines.

As for the statistical analysis, the data were weighted using the following question to measure the network size: “How many female sex workers do you know personally?.” In each municipality, the expansion factors were inversely proportional to the network size.^[22]^

The research project was approved by the Ethics Committee of the FIOCRUZ (Case no. 395/07).

### 2.2 The RDS study among FSW, 2016

Information on FSW aged 18 years or over were collected in 2016 in 12 Brazilian cities, a priori chosen by the DDAHV according to both, geographical criteria and the epidemiologic relevance of the HIV/AIDS epidemic in the country. The minimum sample size was set at 350 FSW in each city (Table 1). Women were eligible to participate in the study with the same inclusion criteria as the baseline study.

Fieldwork was conducted in health services located in the 12 cities. For each site, 6 to 8 seeds were chosen purposively, following formative qualitative research. Each seed received 3 coupons to distribute to other sex workers within her social network. After participating in the interview, each participant received 3 additional coupons to distribute to their peers and this process was repeated until the sample size was achieved in each site. Similar to 2009, primary (makeup products and reimbursement) and secondary incentives (approximately US$10.00 for recruitment) were given to each participant.

The questionnaire included the same modules applied in the baseline study, with some updates related to new prevention strategies, such as pre- and postexposure prophylaxis (PrEP and PEP). The questionnaire was designed for tablets and could be self-administered according to the willingness and capacity of the participant. Tests for HIV, syphilis, and hepatitis B and C were conducted by standard rapid tests using peripheral venous blood collection, according to protocols recommended by the Brazilian MoH. All tests occurred before the interview and all participants received pre- and posttest counseling. Participants who tested positive in any one of the rapid tests received additional post-test counseling, both to address the psychological impact and to encourage partner notification, and were also referred to public health services for follow-up.

The research project was approved by the Ethics Committee of the FIOCRUZ (Protocol 1.338.989).

### 2.3 Data analysis

The rationale for the data analysis in both studies was to use statistical methods appropriate for data collected with complex sampling design. The analyses took into account the dependence among observations, resulting from the recruitment chains, and the unequal probabilities of selection, resulting from the different network sizes of each participant. The samples were treated as stratified, with clusters, and with different selection probabilities. Each one of the municipalities composed a stratum and, in each one, the weighting was inversely proportional to the size of the network totaling the size of the stratum.^[19]^

In order to take into account homophily—that is, the tendency of a participant to recruit peers with similar characteristics^[23]^ and a potential overrepresentation of individuals with certain characteristics in the study population, the 3 participants invited by the same recruiter were considered a cluster.^[19]^

In the present study, we compared the sample distributions obtained in the 2009 and 2016 RDS based on the comparison 95% confidence intervals for each estimate. The following characteristics were analyzed: Sociodemographic variables (age group; educational level; and race); commercial sex characteristics, such as type of work venue, price of the sexual encounter, starting age of sex work; frequency of drug use; participation in prevention activities (affiliated to/or participated in a nongovernmental organization (NGO) in defense of FSW rights; received condoms free of charge, and STI counseling in the past 6 months); history of HIV and syphilis testing; frequency of Pap smear examination; discrimination in health services; sexual behavior with steady partners and clients. Additionally, changes in HIV and syphilis prevalence were also analyzed.

## 3 Results

In Table 1, we show the different sample compositions in 2009 and 2016 studies, for the study municipalities as well as for the sample sizes in each municipality. The average number of recruitments was 2.2, in 2009, and 2.3, in 2016, with small variation among sites.

Results of Table 2 show similar sample distributions by race and age group, with a slightly higher proportion of FSW 40 years old or over in 2016, while the proportion of FSW with better educational level was higher in 2016.

**Table 2:**
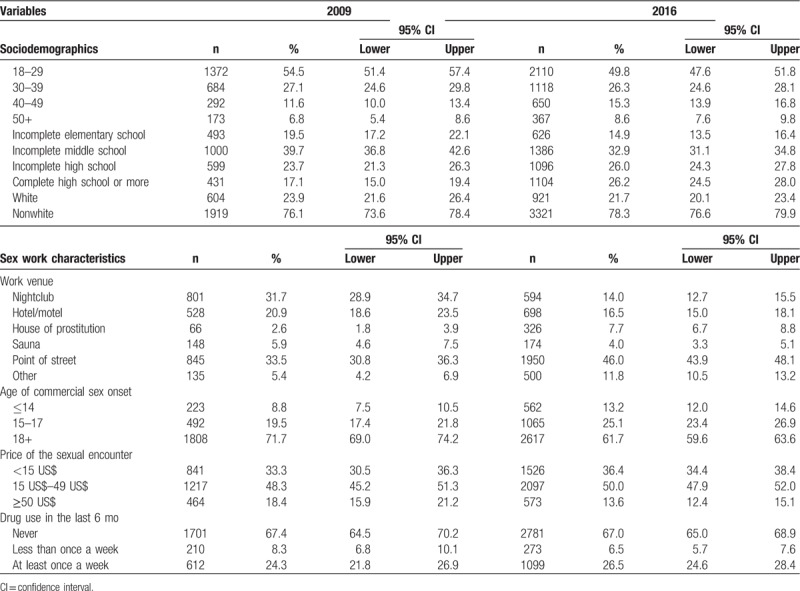
Sample distributions by sociodemographics variables, sex work characteristics, and use of illicit drugs (Brazil, FSW studies, 2009 and 2016).

As to commercial sex characteristics, there was a higher proportion of street FSW in 2016 (46.0%) as compared to 2009 (33.5%), while the reverse was seen for those who worked in nightclubs (14.0% and 31.7% for 2016 and 2009, respectively). The price of sexual encounter did not show statistically significant change. However, commercial sex debut shifted to younger ages: while in 2009 the proportion of women who started sex work under 18 years old was 28.3%, in 2016 this percentage rose to 38.3%. Also, the proportion of girls who started sex work even earlier (under 14 years old) increased from 8.8% to 13.2%, from 2009 to 2016 (Table 2). There were no differences in the proportions of drug use between the periods, and the use of illicit drugs at least once a week also remained at approximately 25%.

In Table 3, results indicate that the already low proportion of FSW affiliated to an NGO in defense of their rights (14.0%), in 2009, further decreased to 7.8%, in 2016. Although, there was some evidence of an increase in free condom distribution from 77.2%, in 2009, to 81.8%, in 2016, the proportion of FSW who received counseling on STI in the past 6 months was significantly reduced from 47.5%, in 2009, to 24.4%, in 2016. However, in 2016, the quantity was considered sufficient for more than 70% among those who received free condoms (Table 3).

**Table 3:**
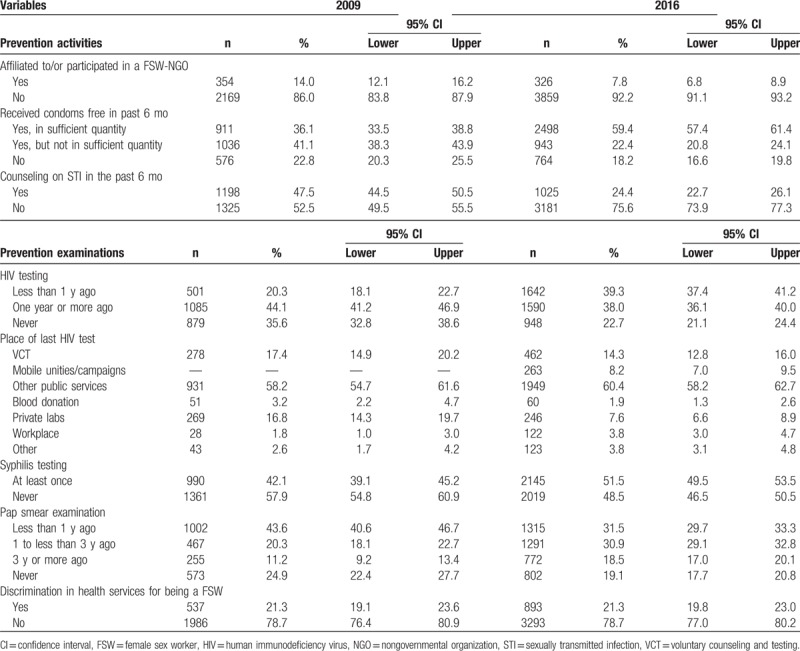
Sample distributions by participation in prevention activities, history of HIV and syphilis testing, Pap smear examination, and discrimination in health services (Brazil, FSW studies, 2009 and 2016).

Some progress was found for HIV testing. As shown in Table 3, the proportion of FSW who had been tested in the last 12 months increased from 20.3% to 39.3%, and the proportion of FSW who had never been tested decreased from 35.6% to 22.7%. Also, the proportions of those who were never tested for syphilis dropped from 57.9%, in 2009, to 48.5%, in 2016 (Table 3). However, an opposite decreasing trend was found for the Pap smear examination. The proportion of a Pap smear examination in the last 12 months decreased from 43.6%, in 2009, to 31.5%, in 2016. At the same time, perception of discrimination in health services remained at similar levels. In both studies, more than one-fifth of the participants felt discriminated against in health services for being a FSW.

Table 4 results show improvements in safe sex with clients. Regular condom use significantly increased from 2009 to 2016. For example, the proportion of regular condom use with clients in vaginal sex increased from 69.9% to 80.5%. With steady partners, however, the improvement is unclear: although regular condom use rose from 2009 to 2016, the proportion of never using condoms also increased.

**Table 4:**
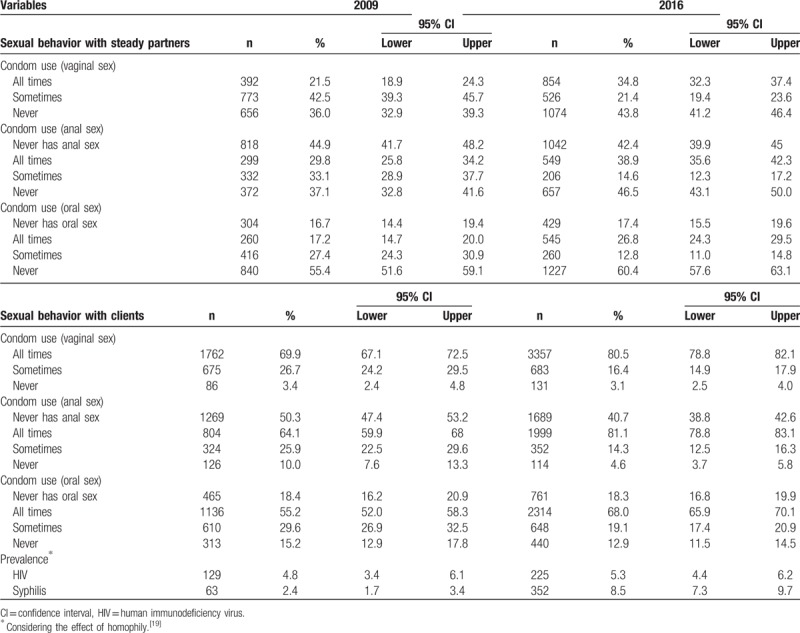
Sample distributions by sexual behavior with steady partners and with clients and prevalence of HIV and syphilis (Brazil, FSW studies, 2009 and 2016).

**Figure 1:**
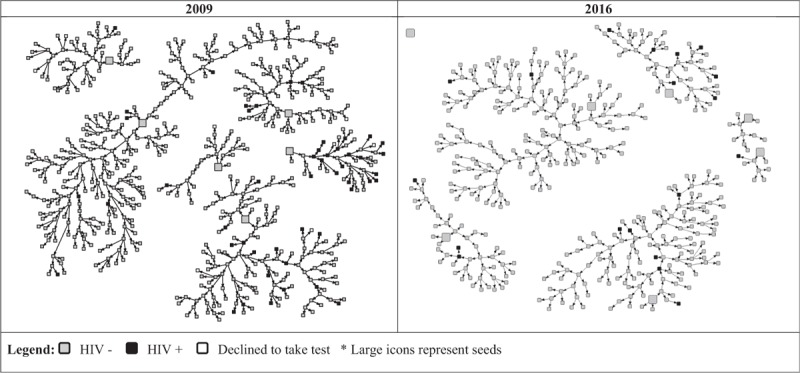
Rio de Janeiro networks. FSW studies, 2009 and 2016.

As to HIV prevalence, the 5% level was sustained and no significant differences were found from 2009 to 2016. However, syphilis prevalence was found to be more than 3 times higher in 2016 (8.5%) than in 2009 (2.4%). Effects of homophily for HIV infection were evidenced in both studies, as exemplified for Rio de Janeiro (Fig. 1).

## 4 Discussion

In this article, we compare the results obtained from an RDS study among FSW carried out in 2016 with the findings of the baseline investigation conducted in 2009. Analysis for the overall aggregated data for Brazil shows consistency of the 2 RDS samples. Despite the different set of municipalities and distinct sample sizes by municipality, after weighting, the samples were similarly distributed by age group and race. Furthermore, the higher educational level in 2016 is certainly reflecting the recent improvements in education in Brazil.

As to commercial sex characteristics, it is of concern the increase in the proportion of adolescents who started commercial sex under 18 years old, with 13% starting at age 14 years old or younger. Although prostitution is not an illegal occupation in Brazil, sexual exploitation of minors is criminalized by the Brazilian legislation.^[14]^ Consequences of sexual exploitation of minors are reflected in the marginalization of female adolescents from health policies. Previous studies in Brazil have already emphasized the need of ongoing STI prevention activities among sexually active female adolescents.^[24,25]^

Some advances have been achieved in distributing free condoms. The study showed not only an increase in proportion of FSW receiving condoms free of charge, but also a substantial increase in the proportion that considered the amount satisfactory. The expansion in condom distribution has already shown impact on safe sex practices. The regular use of condoms with clients rose considerably from 2009 to 2016.

On the other hand, the percentage of women who have participated in NGOs in defense of FSW rights is decreasing in Brazil and is currently at a low 8% level. Admittedly, the participation of the civil society was a key element to promote human rights, contributing to the Brazilian response to the HIV/AIDS epidemic.^[26]^ Nevertheless, with the reduction in funding, there has been a significant decrease in the number of NGOs and activists dedicated to most at risk populations. Findings of this comparison analysis already reflect the consequences, such as the reduction of community-based counseling and health promotion.

In the last 30 years, Brazil has undergone several changes in terms of health care. The country has evolved to a unified health system with profound changes in health care policies and a marked expansion of primary health care.^[27]^ More recently, several initiatives were undertaken focused on increasing cervical cancer screening as well as HIV, syphilis and hepatitis testing in primary care services.^[28,29]^ In fact, the results of this study confirm the improvements in HIV testing and the use of primary care units as the main source of HIV testing among FSW, with a decreasing proportion seeking voluntary counseling and testing (VCT) units and private labs.

Other initiatives to encourage HIV testing have been introduced in the country. These include mobile unities (trailers) and collaboration with NGOs for stimulating HIV testing, known as “Keep track” (“Fique sabendo”) program.^[30]^ Comparison with the baseline study already shows the impact of these initiatives. HIV testing in the last 12 months increased almost 20%. As the MoH currently has in place the policy of providing antiretroviral therapy to all individuals diagnosed with HIV, the improvement in the frequency of HIV testing is particularly relevant.^[31]^

However, although cervical cancer screening is also available free of charge in primary healthcare units, the frequency of Pap smear screening did not increase from 2009 to 2016 and even showed a decreasing trend in this period. Perhaps because the importance of taking a gynecologic examination is not being sufficiently emphasized in health services. A recent study among FSW in China shows that only 15.3% had a lifetime Pap smear and specifically emphasizes the need of cervical cancer and human papillomavirus (HPV) awareness programs in addition to regular screening for these vulnerable women.^[32]^

At the same time, the results of this study indicate a large and stable discrimination perception in health services for being a sex worker without any further improvement in this aspect. As has been pointed out before, stigma and discrimination within health services constitute one of the main barriers to STI control.^[33]^ Moreover, the anticipation of stigma related to their sexual work, and possibly discrimination by health staff, may affect the disclosure of FSW status in health services.^[34]^ However, if FSW do not reveal their status, primary care physicians would not be aware that they fall into the category of most-at-risk group and would not offer the available interventions.^[35]^

Although a more satisfactory result was found for HIV prevalence, which was sustained at the previous level of 5%, the rise in syphilis prevalence, increasing from 2.5% to 8.4% in 7 years, is alarming. Additionally, the numbers of diagnosed cases of congenital syphilis are on the rise as well.^[36]^ During the last 10 years, a resurgence of syphilis has occurred in many other countries.^[37]^ In the United States, 2014 data from the CDC indicate a sharp increase in the rate of new cases of syphilis, predominantly in the most vulnerable groups.^[38]^

Many are the challenges to be faced in attempting to reverse the upward trend of syphilis among FSW in Brazil. Despite the progress in condom distribution free of charge, it is necessary to increase awareness campaigns, emphasize the use of condom, reaffirm the importance of STI counseling, and reiterate the need of regular syphilis screening in this key population group. Due to the difficulties of discussing sexual behavior or disclosing their FSW status in health services, risky practices may not be appropriately addressed. In this context, there is a need to expand access to public health services and provide preventive screening for all sexually active women, regardless of age or occupation.

## Acknowledgments

The authors would like to express their gratitude to the participants of the study and to the local teams that carried out the fieldwork in the 12 cities. We are also grateful for the support of STI/HIV/Aids and Viral Hepatitis Department of the Brazilian Minister of Health. Additionally, we appreciate the support of The Brazilian FSW Group: Célia Landmann Szwarcwald, Paulo Roberto Borges de Souza Júnior, Orlando C. Ferreira Jr., Giseli Nogueira Damacena, Neide Gravato da Silva, Rita Bacuri, Helena Brigido, Hermelinda Maia Macena, Ana Brito, Inês Dourado, Mark Drew Crosland Guimarães, Wanessa da Silva de Almeida, Alexandre Grangeiro, Carla Luppi, Karin Regina Luhm, Isete Maria Stella, Adriana Varela Espinola, Tânia Varela, Francisca Sueli da Silva.

## Author Contributions

All authors contributed to the concept of the paper and data analysis. CLS and MDCG were responsible for the writing of the final version of the manuscript. WSA, GND and PRBSJ were responsible for the statistical analyses. OCFJ was responsible for HIV and syphilis testing. All authors have read and approved the paper.

## References

[1] Barbosa-Jr A Pascom ARP Szwarcwald CL Transfer of sampling methods for studies on most-at-risk populations (MARPs) in Brazil. Cad Saude Publica 2011;27:S36–44. 21503522 10.1590/s0102-311x2011001300005

[2] Grassly NC Garnett GP The future of the HIV pandemic. Bull World Health Organ 2005;83:378–82. 15976879 PMC2626235

[3] Spire B Zoysa I Himmich H HIV prevention: what have we learned from community experiences in concentrated epidemics? J Int AIDS Soc 2008;11:5. 19014656 10.1186/1758-2652-11-5 PMC2584058

[4] Boily MC Lowdes C Alary M The impact of HIV epidemic phases on the effectiveness of core group interventions: insights from mathematical models. Sex Transm Infect 2002;78:i78–90. 12083451 10.1136/sti.78.suppl_1.i78 PMC1765829

[5] Mishra S Steen R Gerbase A Impact of high-risk sex and focused interventions in heterosexual HIV epidemics: a systematic review of mathematical models. PLoS ONE 2012;7:e50691. 23226357 10.1371/journal.pone.0050691 PMC3511305

[6] Ramanathan S Nagarajan K Ramakrishnan L Inconsistent condom use by male clients during anal intercourse with occasional and regular female sex workers (FSWs): survey findings from southern states of India. BMJ Open 2014;4:e005166. 10.1136/bmjopen-2014-005166 PMC4244455 25410604

[7] Carael M Slaymaker E Lyerla R Clients of sex workers in different regions of the world: hard to count. Sex Transm Infect 2006;82:iii26–33. 16735289 10.1136/sti.2006.021196 PMC2576731

[8] Lowndes CM Alary M Labbé AC Interventions among male clients of female sex workers in Benin, West Africa: an essential component of targeted HIV preventive interventions. Sex Transm Infect 2007;83:577–81. 17942573 10.1136/sti.2007.027441 PMC2598661

[9] Semaan S Lauby J Liebman J Street and network sampling in evaluation studies of HIV risk-reduction interventions. AIDS 2002;4:213–23. 12555695

[10] Magnani R Sabin K Saidel T Review of sampling hard-to-reach and hidden populations for HIV surveillance. AIDS 2005;19:S67–72. 10.1097/01.aids.0000172879.20628.e1 15930843

[11] Pascom ARP Szwarcwald CL Barbosa-Jr A Sampling studies to estimate the HIV prevalence rate in female commercial sex workers. Braz J Infec Dis 2010;14:385–97. 20963326

[12] Sabin KM Johnston LG Epidemiological challenges to the assessment of HIV burdens among key populations: respondent-driven sampling, time-location sampling and demographic and health surveys. Curr Opin HIV AIDS 2014;9:101–6. 24464090 10.1097/COH.0000000000000046

[13] Brasil. Ministério da Saúde. Secretaria de Vigilância em Saúde. Departamento de DST, Aids e Hepatites Virais. Pesquisa de conhecimento, atitudes e práticas na população brasileira/Ministério da Saúde. Secretaria de Vigilância em Saúde. Departamento de DST, Aids e Hepatites Virais. Brasília: Ministério da Saúde, 2016. 166p.

[14] Brazilian Criminal Code. Number 12984. 2014. Retrieved from http://www.planalto.gov.br/ccivil_03/_Ato2011–2014/2014/Lei/L12984.htm. Accessed August 10, 2017.

[15] Kerr LR Mota RS Kendall C HIV among MSM in a large middle-income country. AIDS 2013;27:427–35. 23291540 10.1097/QAD.0b013e32835ad504

[16] Damacena GN Szwarcwald CL Barbosa-Jr A Implementation of respondent-driven sampling among female sex workers in Brazil, 2009. Cad Saúde Pública 2011;27:S45–55. 21503524 10.1590/s0102-311x2011001300006

[17] Toledo L Codeço CT Bertoni N Putting respondent-driven sampling on the map: insights from Rio de Janeiro, Brazil. J Acquir Immune Defic Syndr 2011;57:S136–43. 21857309 10.1097/QAI.0b013e31821e9981

[18] Heckathorn DD Respondent-driven sampling: a new approach to the study of hidden populations. Soc Probl 1997;44:174–99.

[19] Szwarcwald CL Souza PRB Jr Damacena GN Analysis of data collected by RDS among sex workers in 10 Brazilian cities, 2009: estimation of the prevalence of HIV, variance, and design effect. J Acquir Immune Defic Syndr 2011;57:S129–35. 21857308 10.1097/QAI.0b013e31821e9a36

[20] Damacena GN Szwarcwald CL Souza PRB Jr Risk factors associated with HIV prevalence among female sex workers in 10 Brazilian cities. J Acquir Immune Defic Syndr 2011;57:S144–52. 21857310 10.1097/QAI.0b013e31821e9bf6

[21] Damacena GN Szwarcwald CL Souza PRB Jr HIV risk practices by female sex workers according to workplace. Rev Saude Publica 2014;48:428–37. 25119937 10.1590/S0034-8910.2014048004992 PMC4203086

[22] Salganik MJ Heckathorn DD Sampling and estimation in hidden populations using Respondent-Driven Sampling. Sociol Methodol 2004;34:193–240.

[23] Heckathorn DD Respondent-Driven Sampling II: deriving valid population estimates from chain-referral samples of hidden populations. Soc Probl 2002;49:11–34.

[24] Miranda AE Szwarcwald CL Peres RL Prevalence and risk behaviors for chlamydial infection in a population-based study of female adolescents in Brazil. Sex Transm Dis 2004;31:542–6. 15480115 10.1097/01.olq.0000137899.25542.75

[25] Guimarães EM Guimarães MD Vieira MAS Lack of utility of risk score and gynecological examination for screening for sexually transmitted infections in sexually active adolescents. BMC Med 2009;7:8. 19284575 10.1186/1741-7015-7-8 PMC2664828

[26] Gómez EJ Harris J Political repression, civil society and the politics of responding to AIDS in the BRICS nations. Health Policy Plan 2016;31:56–66. 25858965 10.1093/heapol/czv021

[27] Paim J Travassos C Almeida C The Brazilian health system: history, advances, and challenges. Lancet 2011;377:1778–97. 21561655 10.1016/S0140-6736(11)60054-8

[28] Barcelos MRB Lima RCD Tomasi E Quality of cervical cancer screening in Brazil: external assessment of the PMAQ. Rev Saude Publica 2017;51:67. 28746576 10.1590/S1518-8787.2017051006802 PMC5510783

[29] Pereira GFM Sabidó M Caruso A Transitioning from antenatal surveillance surveys to routine HIV testing: a turning point in the mother-to-child transmission prevention programme for HIV surveillance in Brazil. BMC Infect Dis 2017;17:469. 28679418 10.1186/s12879-017-2540-4 PMC5499045

[30] Szwarcwald CL Damacena GN Miranda RL HIV testing among men in Curitiba, Brazil. AIDS Care 2018;30:56–8. 28934867 10.1080/09540121.2017.1363370 PMC6501198

[31] Montaner JS Lima VD Harrigan PR Expansion of HAART coverage is associated with sustained decreases in HIV/AIDS morbidity, mortality and HIV transmission: the “HIV Treatment as Prevention” experience in a Canadian setting. PLoS ONE 2014;9:e87872. 24533061 10.1371/journal.pone.0087872 PMC3922718

[32] Hong Y Zhang C Li X HPV and cervical cancer related knowledge, awareness and testing behaviors in a community sample of female sex workers in China. BMC Public Health 2013;13:696. 23898889 10.1186/1471-2458-13-696 PMC3733604

[33] Beattie TSH Bhattacharjee P Suresh M Personal, interpersonal and structural challenges to accessing HIV testing, treatment and care services among female sex workers, men who have sex with men and transgenders in Karnataka state, South India. J Epidemiol Community Health 2012;66:ii42–8. 22495772 10.1136/jech-2011-200475

[34] Pescosolido BA Martin JK The stigma complex. Annu Rev Sociol 2015;41:87–116. 26855471 10.1146/annurev-soc-071312-145702 PMC4737963

[35] Nyblade L Reddy A Mbote D The relationship between health worker stigma and uptake of HIV counseling and testing and utilization of non-HIV health services: the experience of male and female sex workers in Kenya. AIDS Care 2017;29:1364–72. 28325068 10.1080/09540121.2017.1307922

[36] Saraceni V Pereira GFM Silveira MF [Epidemiological surveillance of vertical transmission of syphilis: data from six federal units in Brazil]. Rev Panam Salud Publica 2017;41:e44 Portuguese. 28614467 10.26633/RPSP.2017.44 PMC6612729

[37] Halatoko WA Landoh DE Saka B Prevalence of syphilis among female sex workers and their clients in Togo in 2011. BMC Public Health 2017;17:219. 28222772 10.1186/s12889-017-4134-x PMC5320666

[38] Willeford WG Bachmann LH Syphilis ascendant: a brief history and modern trends. Trop Dis Travel Med Vaccines 2016;2:20. 28883964 10.1186/s40794-016-0039-4 PMC5530970

